# Designing a handwashing station for infrastructure-restricted communities in Bangladesh using the integrated behavioural model for water, sanitation and hygiene interventions (IBM-WASH)

**DOI:** 10.1186/1471-2458-13-877

**Published:** 2013-09-23

**Authors:** Kristyna RS Hulland, Elli Leontsini, Robert Dreibelbis, Leanne Unicomb, Aasma Afroz, Notan Chandra Dutta, Fosiul Alam Nizame, Stephen P Luby, Pavani K Ram, Peter J Winch

**Affiliations:** 1 Social and Behavioral Interventions Program, Department of International Health, Johns Hopkins Bloomberg School of Public Health, Baltimore, MD, USA; 2 Water, Sanitation and Hygiene Research Group, Centre for Communicable Disease, International Centre for Diarrhoeal Disease Research Bangladesh, Dhaka, Bangladesh; 3 Department of Social and Preventive Medicine, School of Public Health and Health Professions, University at Buffalo, Buffalo, USA; 4 Woods Institute for the Environment, Stanford University, Stanford, USA

**Keywords:** formative research, Qualitative methods, Trials of improved practices, Handwashing, Hygiene behaviour, Behaviour change, Behavioural model, Handwashing technology, Enabling technology, Handwashing station, Soapy water, Feasibility, Acceptability

## Abstract

**Background:**

In Bangladesh diarrhoeal disease and respiratory infections contribute significantly to morbidity and mortality. Handwashing with soap reduces the risk of infection; however, handwashing rates in infrastructure-restricted settings remain low. Handwashing stations – a dedicated, convenient location where both soap and water are available for handwashing – are associated with improved handwashing practices. Our aim was to identify a locally feasible and acceptable handwashing station that enabled frequent handwashing for two subsequent randomized trials testing the health effects of this behaviour.

**Methods:**

We conducted formative research in the form of household trials of improved practices in urban and rural Bangladesh. Seven candidate handwashing technologies were tested by nine to ten households each during two iterative phases. We conducted interviews with participants during an introductory visit and two to five follow up visits over two to six weeks, depending on the phase. We used the Integrated Behavioural Model for Water, Sanitation and Hygiene (IBM-WASH) to guide selection of candidate handwashing stations and data analysis. Factors presented in the IBM-WASH informed thematic coding of interview transcripts and contextualized feasibility and acceptability of specific handwashing station designs.

**Results:**

Factors that influenced *selection* of candidate designs were market availability of low cost, durable materials that were easy to replace or replenish in an infrastructure-restricted and shared environment. Water storage capacity, ease of use and maintenance, and quality of materials determined the acceptability and feasibility of specific handwashing station designs. After examining technology, psychosocial and contextual factors, we selected a handwashing system with two different water storage capacities, each with a tap, stand, basin, soapy water bottle and detergent powder for pilot testing in preparation for the subsequent randomized trials.

**Conclusions:**

A number of contextual, psychosocial and technological factors influence use of handwashing stations at five aggregate levels, from habitual to societal. In interventions that require a handwashing station to facilitate frequent handwashing with soap, elements of the technology, such as capacity, durability and location(s) within the household are key to high feasibility and acceptability. More than one handwashing station per household may be required. IBM-WASH helped guide the research and research in-turn helped validate the framework.

## Background

### Introduction

Poor water quality and hygiene are major contributors to the spread of diarrhoeal disease and acute respiratory infections (ARI). Estimates suggest that in 2011 there were 1.3 million ARI-related deaths and 700,000 diarrhoea-related deaths among children under the age of five [1]. Handwashing with soap has been shown to significantly reduce the incidence of both respiratory infections and diarrhoea. In a meta-analysis of three systematic reviews, handwashing with soap was shown to reduce risk of diarrhoeal disease by 48 per cent [2], and another meta-analysis showed reduced risk of respiratory infections by 21 per cent [3].

Based on a review of formative research activities from eleven countries, Curtis et al. organized factors that affect handwashing behaviour into two categories: environmental and “brain” factors [4]. Environmental factors include social, physical and biological influences that shape handwashing behaviours. Physical factors, in particular, include the cost of soap, water, and access to handwashing stands [4,5]. In Kenya, Schmidt et al. [6] found that “structural constraints” – such as access to water inside, rather than outside, the home - influences the likelihood of handwashing at key times. Findings from Zimbabwe suggest that altering norms and community structures in support of positive hygiene behaviours can have a positive impact on behavioural outcomes [7].

The “brain” – or cognitive – factors that influence behaviours include an individual’s habits, planning and motivation for behavioural change [4]. Research in Kenya found that study participants reporting a higher degree of handwashing habit and past experience with handwashing had improved handwashing practices during structured observations compared to those who reported less-developed habits [8]. Past behaviours along with motivational factors such as disgust, social concerns, and feelings of being a good mother were found to influence individual handwashing practices in Ghana [9]. Planned behaviours – those behaviours intended to avoid a specific negative health outcome – are often associated with health and disease knowledge. Studies have demonstrated an increase in handwashing practices with improved knowledge of key moments for handwashing [10].

A handwashing station may facilitate behaviour by providing soap and water together in an established location convenient to the behaviour, such as near a toilet or in a food preparation area [5,11]. In addition to establishing a designated place for handwashing, the design of the handwashing station influences use. The Water and Sanitation Program’s (WSP) Global Scaling Up Handwashing Project has compiled a database of handwashing station designs and enabling technologies [12] and links the physical attributes of products to behaviour [13]. Devine reported that characteristics, such as tap design, soap presentation, and container parameters, influenced acceptability of the handwashing station in Vietnam [14].

The purpose of this study was to inform the design of a handwashing station for two subsequent randomized-controlled trials (RCTs) in Bangladesh testing the health effects of handwashing. Data on handwashing and hygiene practices in the country showed that there was considerable room for improvement: in a population-based, cross-sectional survey, only 14% of people washed both hands with soap after defecation during five hours of structured observation [15]. In a sample of over 1,000 villages in rural Bangladesh, 72% of households had access to water after using the toilet, yet only 42% had access to soap as well [11]. Having both soap and water available at the place for handwashing after toileting was associated with a two-fold increase in handwashing with soap after faecal contact [16].

## Methods

### Guiding theoretical framework

**Figure 1:**
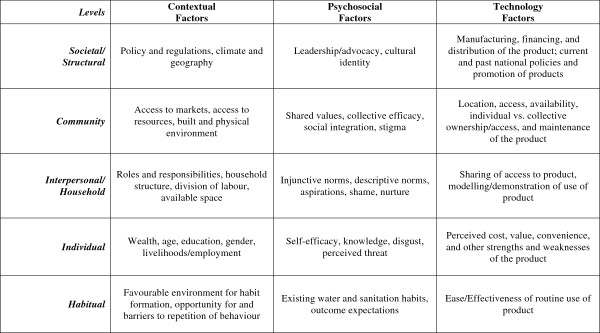
**Integrated behavioural model for water, sanitation and hygiene (IBM-WASH).** The IBM-WASH outlines key factors that influence behaviours in water, sanitation and hygiene interventions. These behavioural factors are organized by cell, suggesting the level and domain (Contextual, Psychosocial, Technology) in which factors operate (Dreibelbis et al. submitted for publication).

The Integrated Behavioural Model for Water, Sanitation and Hygiene interventions or IBM-WASH [17], outlined in Figure 1, presents a synthesis of behavioural models related to water, sanitation, and hygiene (WASH) and organizes factors that affect behaviour in an ecological framework. The IBM-WASH identifies three dimensions Contextual Factors (e.g., access to water and soap), Psychosocial Factors (e.g., disgust related to contact with unclean objects, perceived risk of disease, pre-existing habit, etc.), and Technological Factors (i.e., related to the physical hardware storing soap and water) – each of which function at five aggregate levels: habitual, individual, interpersonal/household, community and societal/structural [17].

We used the factors outlined in the IBM-WASH to guide selection of candidate designs and qualitative data analysis. Factors represented in the IBM-WASH framework informed thematic coding of interview transcripts, and contextualized feasibility and acceptability of specific handwashing station designs. In turn, the results of this research process helped validate the application of the IBM-WASH framework to a specific technology-supported behavioural outcome.

### Definition and selection of candidate handwashing technologies and soap formulations

We defined a handwashing station as a dedicated, convenient location where both soap and water are available for handwashing. We considered the contextual factors at the societal/structural and community levels of the IBM-WASH framework during technology selection (Figure 1). Because the subsequent intervention was intended for use in resource-constrained settings, candidate handwashing stations were low-cost (between USD 0.07 and 6.50), simple technologies made with few moving parts, self-contained, and did not require electricity for operation. Parts and supplies needed to be easily replaceable from existing materials in the household or easily purchased locally.

Soap was valuable, scarce, and often stored far away from the water to ration its use. During initial direct observations/transect walks in potential study areas, we observed that some households were using soapy water in a bottle placed near their water source instead of bar soap, in order to minimize waste and prevent soap theft. Soapy water was therefore included as a candidate soap formulation, either as part of a handwashing station design, or to convert the household’s water source into a complete handwashing station when placed nearby.

**Figure 2:**
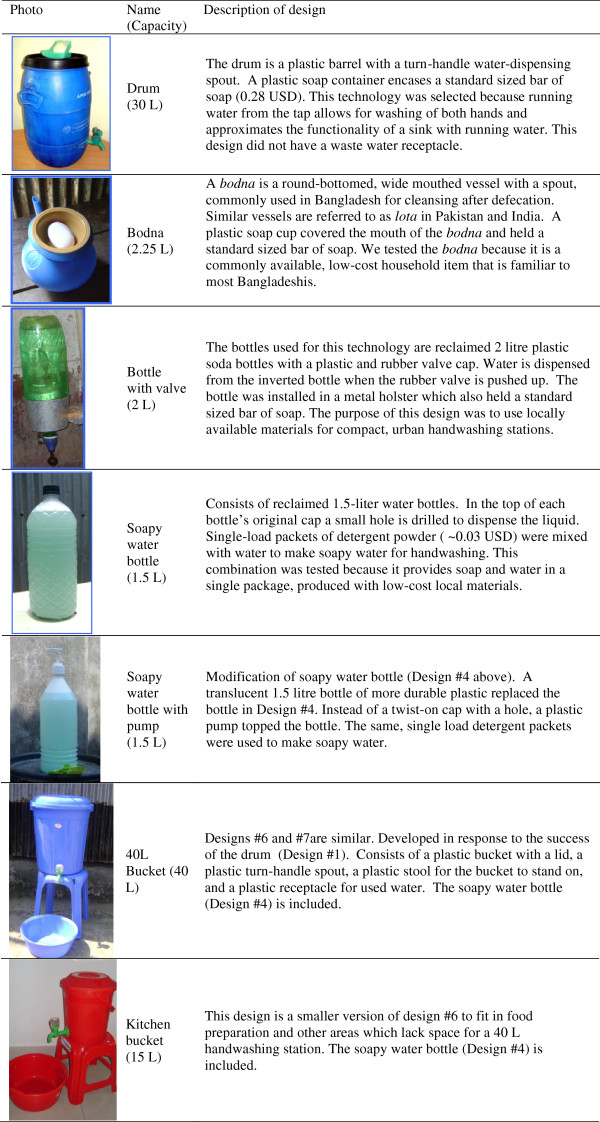
Evaluated handwashing technology designs.

We tested a total of seven handwashing station designs in two phases (see Trials of improved practices below for details on phases). Details on each of the following designs are presented in Figure 2:

Phase 1 designs:

• 30 Litre drum with tap and soap container

• 2.25 Litre *Bodna* (pot with spout traditionally used for anal cleansing after toileting) with soap cup

• 2 Litre Bottle (water only) with a valve cap and soap container

• 1.5 Litre Soapy water bottle with a hole in the cap for dispensing (placed at the water source)

Phase 2 designs:

• 1.5 Litre Soapy water bottle with pump (placed at the water source)

• 40 Litre Bucket with tap, 10 L basin, stool used as a stand, and soapy water bottle

• 15 Litre Kitchen bucket with tap, 8 L basin, stand, and soapy water bottle

Of the seven candidate designs, five were tested in the urban field site, five in the rural field site, and three designs were tested at both sites.

### Study area

Mohammadpur, a sub-district (*thana*) of metropolitan Dhaka, served as our urban field site. The population resided in densely populated compounds (*bashas)* consisting of ten or more families in semi-permanent brick wall rooms with tin roofs. Households did not necessarily share familial ties and often shared access to cooking areas, outside water sources, and sanitation facilities with neighbouring households. The rural study site included households from Kishoreganj, a district in central Bangladesh northeast of Dhaka. Households that shared familial relations were clustered into compounds (*baris*). Households typically shared a common courtyard with access to outside water source and latrines and usually had their own cooking facilities. Each site was selected as representative of infrastructure-restricted, low-income, urban or rural residence with no or minimal household-level water and sanitation access.

### Selection of study households

In both study sites we prepared a list of all compounds within each study area. In the urban site (Mohammadpur), we selected ten compounds through a systematic sample of every fifth compound (with a random start) to minimize intervention contamination. Within selected compounds, we recruited households with at least one child under the age of five for participation in the trials. We identified an average of five households per compound that met our selection criteria for a total of 50 participating households. In the rural site (Kishoreganj), we identified three clusters of compounds from different geographic areas of the village to minimize intervention contamination. Each cluster had ten compounds and an average of one household per compound that met our selection criteria of having a child under the age of five for a total of 29 participating households. A socio-economic and demographic profile of the study participants is provided in Table 1.

**Table 1:** Demographic and socio-economic profile of study participants by site

		**Urban**	**Rural**
		n = 50*	**n = 29**
**Age**			
	≤25 years	20	10
	26-34 years	14	7
	35-44 years	7	4
	45 to	5	8
**Sex**			
	Male	7	6
	Female	39	29
**Educational level**			
	Illiterate	10	6
	Can sign	2	5
	≤Grade 5	18	5
	Grade6-10	15	12
	Upper grade 10	1	1
**# of HH members**			
	≤ 5	33	16
	5>	13	13
**Profession**			
	Housewife	33	22
	Agriculture	-	6
	Small business	4	-
	Service	2	-
	Maid/servant	3	-
	Other	4	1

* Data could not be obtained for 4 urban households.

### Trials of improved practices

We tested candidate handwashing stations using trials of improved practices (TIPs), a formative research methodology. TIPs are used to assess the feasibility and acceptability of candidate improved behaviours [18]. The TIPs methodology has been used to inform a range of behavioural interventions including the development of infant feeding schemes [19], increased bed net usage [20], and improved corrals and corralling practices to reduce transmission of diarrhoeal disease associated with domestic animal husbandry [21].

Candidate technologies were assessed in two phases (Table 2). Phase 1 was characterized by an iterative testing and design adjustment [14] in which four technologies were tested: drum with tap and soap container, *bodna* with soap, bottle (water only) with valve cap and soap container, and soapy water bottle with cap and hole placed by the water source. Forty of the recruited households in the urban site participated in Phase 1. Based on preliminary feedback on the bottle with valve cap, this design was not tested in the rural areas and all 30 recruited households participated in Phase 1. Households selected handwashing station options by lottery whereby household representatives selected their preferred design in the order in which names were drawn. After ten households selected a design, the design was removed from the list of available options. Following design selection, field research officers visited the corresponding households and installed it at a suitable location in consultation with the family. Research officers demonstrated the design’s use and maintenance and informed about future visits to seek the family’s on-going consultation with regard to feasibility and acceptability based on experience with actual use.

**Table 2:** Sites and phases of testing for handwashing station designs

**Study site**	**Phase**	**Handwashing technology**	**# households evaluating the design**	**# of follow-up visits per household**
Mohammadpur (urban) 50 households*	1	Drum with tap and soap container	10	5
		*Bodna* with soap cup	10	5
		Bottle with valve cap	10	5
		Bottle with soapy water	10	4
	2	Soapy water bottle with pump	10	2
Kishoreganj (rural) 29 households**	1	Drum with soap container	10	5
		*Bodna* with soap cup	10	5
		Bottle with soapy water	9	5
	2	15 L Kitchen bucket with tap	9	3
		40 Litre bucket with tap	10	4

* total number of interviews for the urban site: ~210.

** total number of interviews for the rural site: ~220.

During Phase 1, follow-up semi-structured, qualitative interviews were completed with the participants within the week of installing the handwashing station, and then at days 7, 15, 30 and 45. Interview guides included questions about handwashing (when and where the behaviour was taking place), use of the handwashing station (where the station was located, how often it was used, and preferences toward the design) and barriers or facilitators to use (see Table 3). In addition, participants were consulted on technical issues with the new technology, such as placement of the handwashing station in the compound or household, maintenance, tap functionality, and potential leakage.

**Table 3:** Interview guide

**Question type**	**Sub-question**
General questions regarding handwashing knowledge and practices	1. Describe your handwashing practices? Where and when do you generally wash hands?
	2. Before receiving this handwashing station how did you wash your hands?
Questions relating to handwashing station design	1. What is your reaction to this handwashing station? Does it help to wash your hands? Describe the advantages?
	2. Do you use X handwashing station? After what activities are you most likely to use it?
	3. Do all members of your household wash their hands with handwashing station? Who in the household uses the handwashing station the most and why?
	4. How did your neighbours react to this hand washing station? Were they interested? Did they want one for their own?
	5. Does the handwashing station help your family members? Do they like using it?
Questions identifying issues surrounding use of the handwashing station	1. What problems have you had with the hand washing station?
	2. Was the hand washing station moved from the place it was installation?

Findings from Phase 1 were used to inform the improved designs tried in Phase 2. During Phase 2, the remaining ten recruited urban households which had not yet tested a design were assigned the soapy water bottle with a pump. In the rural site, 19 of the participating households from Phase 1 were assigned either the 40 L bucket with a tap, stand, basin and soapy water bottle with pump, or the 15 L version (Table 2). There was no lottery in Phase 2. Data collection procedures were similar during Phase 2, however, there were fewer follow-up visits and shorter follow-up periods: two follow-up visits in the urban area over a two week period, and three or four follow-up visits in the rural area over a three week period.

### Data analysis

Qualitative data from interview transcripts were translated from Bengali to English. Responses from each household were compiled for each question in the interview guides, and then sorted according to each handwashing station design and study location.

In our analysis we sought to identify key factors making use of a given handwashing station acceptable and feasible. We defined acceptability to include appropriateness and satisfaction with the handwashing station, including an agreement to install, maintain, and use it to regularly wash hands. Feasibility referred to whether a handwashing station design could physically withstand frequent use and that component parts were replaceable, low-cost and locally available.

We analysed interview data according to the three main dimensions (Contextual, Psychosocial, and Technology) and the five levels of the IBM-WASH framework.

In order to code the qualitative data, four researchers analysed a subset each of the compiled responses and coded the transcripts line-by-line to identify key emergent themes. We compared these initial codes to determinants in an early iteration of the IBM-WASH framework. Details of this process of model development and revision are described in Dreibelbis et al. [17]. Using the refined constructs from the final iteration of IBM-WASH, we developed a final codebook for analysis of the interview data. All compiled responses were coded with the IBM-WASH-based codebook using Atlas.ti Version 5.2.

Although the iterative phases were informative for intervention design, we structure our study findings by the IBM-WASH framework, rather than presenting results by phase, to emphasize influential behavioural determinants.

### Informed consent

Informed consent was obtained from adult participants from each household. The study protocol was reviewed and approved by Ethical Review Committee of icddr,b (International Centre for Diarrhoeal Disease Research, Bangladesh).

## Results

We first present key factors of the Technology dimension of the IBM-WASH in detail. We then present key factors of the Psychosocial and Contextual dimensions. Following these results, we present the designs finally selected for use in the subsequent RCTs.

### Technology dimension at the habitual level

At the Habitual level, the two most significant factors affecting routine use of the handwashing stations were ease of use and visual cues. All participants were capable of using their handwashing stations. However, some participants specifically noted aspects of the handwashing technology that made them easy to use. For some, turning a tap and running water over both hands, as opposed to pouring water from one hand to the other, made washing both hands easy. The drum, kitchen bucket and 40 Litre bucket were especially easy to use because the tap created a continuous stream of water while washing.

A few participants expressed that children or elderly members of the household might have difficulty with using the bottle with valve and the *bodna*. A few participants described that pushing or lifting the vessel with one hand to wash the other hand was difficult, especially for young children. If the handwashing station was too difficult to use, caretakers became responsible for helping the old and young to wash hands.

Several of the handwashing stations served at the same time as visual cues or reminders for people to wash hands. Several participants said that seeing the handwashing station at certain times, such as after defecation or before preparing meals, reminded them to wash their hands. One participant mentioned, *“The drum is a reminder to wash hands because it is installed near the toilet”*. And another said, *“This station (bottle with valve cap) acts as reminder for us to wash our hands because it is always in front of us.”*

Placement of the handwashing station influenced the ability of the device to provide visual cues. In most households, keeping the handwashing device at the toilet was too far removed from other household activities to serve as a reminder to wash hands before cooking or eating. In order to address this problem and to provide easy access for handwashing inside the house, we tested the kitchen bucket for use while cleaning, cooking and eating in the home. One rural participant expressed that this increased her handwashing practice, saying,

When I am busy with other work, I would not regularly go to the tubewell [located outside of the house] to clean my hands before food preparation because it is placed far away from where I cook. But now I wash regularly with the kitchen handwashing station before cooking.

### Technology dimension at the individual level

Operating at the Individual level were reactions to the handwashing station, such as attractiveness and quality. Participants testing the bottle with metal valve viewed the reclaimed 2 litre plastic bottle and metal holster as lacking durability and that the design would not withstand frequent use. For others, appearance and perceived value contributed to the acceptability of a handwashing station in the household. Both the 40-litre bucket and the kitchen bucket were brightly coloured, and installed complete with a water receptacle and a stool to place the bucket upon. Users reported that these features made these handwashing stations attractive. Some noted that the water receptacle kept the area surrounding the handwashing station clean and dry, and the stool added stability. For example, one participant said*, “All of my family likes the bucket handwashing station because after washing hands the waste water is stored in the bowl, and the handwashing station doesn’t get muddy underneath.”*

### Technology dimension at the interpersonal / household level

Operating at the Interpersonal / Household level were factors influencing how a handwashing station was used and shared by households and their neighbours.

Because handwashing station designs needed to be refilled with water and soap, the frequency of regular maintenance influenced acceptability of the designs. Handwashing technologies with smaller capacity such as the bottle with valve, *bodna*, or soapy water bottle when used by a large number of people, required frequent refilling and were not conducive to repeated use throughout the day. One participant said, *“The size of the bottle [with pump] is small so we need to refill it frequently, but sometimes we forget.”* Most participants using handwashing stations with small storage capacities acknowledged that it was not feasible to use the handwashing station at all key times—after defecation or cleaning a child’s bottom, and before food preparation or feeding children.

In the urban field site, several participants mentioned concerns regarding shared access to a handwashing station placed next to a shared latrine and the implications this had on maintenance among sharing households. Even with the large storage capacity of the drum, one urban participant said,

In the last few days, when water and soap have run out, I have managed to refill it. But our compound environment is not good. After some time the renters change, so who will take responsibility? Taking care of the soap and water is not possible for everybody. There is no good place to install the drum… [and it] can be broken. Then, quarrels arise. So, single ownership is better.

Not only was use and maintenance difficult to negotiate when the handwashing station was accessible to multiple unrelated households, but theft and damage were also concerns. Several participants using the *bodna*, 40 L bucket, and kitchen bucket said they moved the handwashing station inside at night, so that it would not be stolen. In both urban and rural sites, the large size of the soapy water bottle and the use of inexpensive detergent in the mixture was advantageous compared to bar soap because it was not attractive to steal and was less likely to be misplaced by children.

### Psychosocial dimension

At the Habitual level, past handwashing experiences influenced current attitudes and practices. Many of our participants shared that they were not habituated to using soap and water for handwashing during all key times. However, some participants noted that their newly acquired experience of a designated handwashing station facilitated habit formation. One participant described how her household’s frequent use of the drum changed how they felt about handwashing, *“In the last few days we are becoming habituated to hand washing, and now if we don’t wash our hands then we feel bad.”* Several participants mentioned that though they had past experience with washing hands after defecation, routine use of the handwashing station increased their hygiene habits to include handwashing before cooking and eating.

At the Individual level, knowledge of handwashing station use, self-efficacy, and attitudes toward threats did not significantly distinguish between handwashing station designs, nor seemed to influence handwashing practice. All participants knew how to use their handwashing station. They were fully capable of using the handwashing stations and soap, indicative of self-efficacy. However, some handwashing station designs and locations were preferred or avoided due to feelings of disgust related of defecation. Because *bodnas* are traditionally used for anal cleansing after defecation, using it as a multipurpose handwashing station rendered this design unacceptable in both urban and rural sites. Similarly, placing other handwashing station designs too close to latrines elicited feelings of disgust when used for handwashing prior to food preparation, eating, or feeding a child. This was particularly pronounced in those cases where the handwashing station was in a location accessible to multiple households.

At the Interpersonal / Household level, participants explained how different members of the family interacted with the handwashing stations designs. For example, mothers often described how their children learned to use the handwashing station, suggesting that handwashing was part of a parent’s nurturing role. In addition, participants in both urban and rural sites alluded to descriptive norms for handwashing. Though many lacked established handwashing routines, several participants stated, *“Everybody should wash their hands regularly,”* indicating that some level of hygiene was expected.

### Contextual dimension

Age was an important factor in use of the handwashing station because age often indicated who was in the home and how easy a handwashing station was to use. A child’s age and developmental stage was extremely important in determining acceptability and feasibility. The drum had low acceptability for some participants because children might leave the tap running and muddy the floor. Many participants expressed concerns about children using the soapy water bottle [Phase 1] because it was too heavy for young children to lift and they preferred the modified design with a pump [Phase 2] instead.

At the Interpersonal / Household level, gender roles and responsibilities of household members played a large part in determining handwashing station use and maintenance. Women came in more frequent contact with soap and water for household chores than their male counterparts, were more likely to be in charge of teaching children to wash hands, and were primarily responsible for refilling and maintaining the handwashing station. In both urban and rural sites, several participants noted that men worked and socialized outside of the home, so they did not have access to a handwashing station throughout the day, and their role in maintenance was limited.

At the Community level interviews with participants revealed that the built environment shaped response to handwashing designs. Access to water had a critical impact on functionality of the handwashing station, especially in designs with small water storage capacity. In the urban site, access to water was shared and determined by electrical pumps drawing from a low pressure and intermittent municipal supply. One participant described this problem saying, 

*“In a slum, our hands become dirty the whole day. Moreover, electricity is absent, so water is not available… Water from the* bodna *is finished after one person washes his or her hands.”*

In the urban site, living quarters were small and densely arranged. Finding a convenient location to install a large handwashing station was difficult because living space was at a premium. One participant noted, *“Our mobility inside the room was interrupted due to the installation of the handwashing station because it is congested inside the room.”* This was another reason to test the smaller kitchen bucket.

Lastly, urban households were more likely to be engaged in income generating activities, and have more access to urban markets than rural households. Participants in the urban site often assessed the handwashing station design they had received in terms of availability of replacement parts at the market. One participant said that the soapy water bottle with a pump was not a good option because, *“If it is stolen, we won’t be able to replace it because the pumper is not available.”* Another participant commented on the feasibility of stocking a handwashing station with soap if not provided through the study, saying that soap was expensive and rare in their household.

### Selection of the final handwashing station designs for RCT Pilot testing

Some of the candidate handwashing station designs were eliminated due to significant factors effecting feasibility and acceptability. The bottle with valve was not durable and held small amounts of water; the *bodna* was associated with disgust related to defecation and was deemed inappropriate for other handwashing purposes; and the drum was not very attractive, less stable, and left the area surrounding the handwashing station a muddied mess. Due to the numerous advantages that the soapy water bottle designs presented over bar soap —low cost, easy to make, and not easily stolen, —we included the soapy water bottle as the method of *soap delivery* for use in our final handwashing station designs.

**Figure 3:**
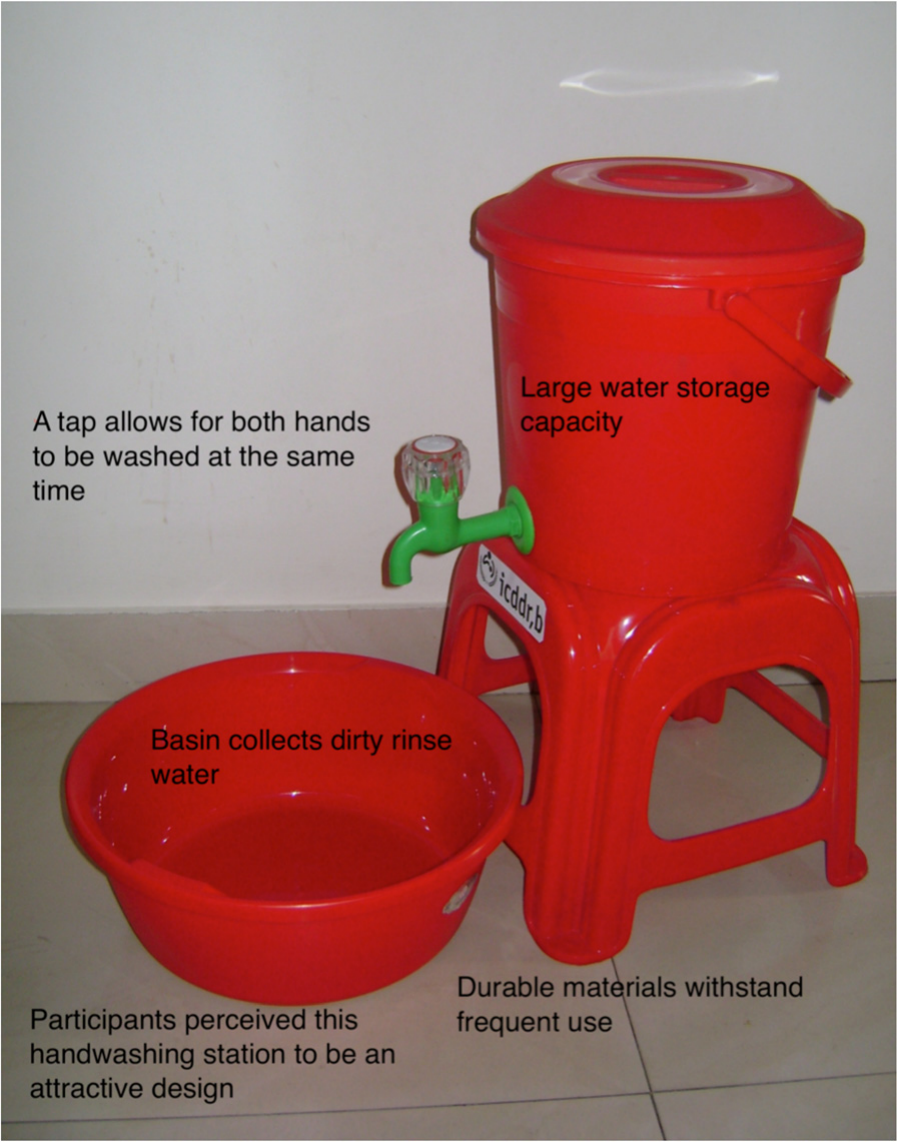
Attributes of a successful handwashing station.

The bucket handwashing stations were also incorporated into our final handwashing station designs (Figure 3). They were attractive, had adequate water storage capacity, and were easy to use. The stand and wastewater receptacle included added to the handwashing stations’ quality. Access to more than one handwashing stations was foreseen as an option for the pilot phase households, in order to facilitate convenience as well as to overcome the problem of disgust associated with any station used for toilet-related handwashing. The 40 litre version was suitable for outdoor use and large indoor spaces associated more with locations near a latrine. The 15 litre version was more suitable for food preparation areas and other tighter spaces.

## Discussion

The study explored factors influencing the feasibility and acceptability of candidate handwashing station designs in two infrastructure-restricted settings in Bangladesh, using the IBM-WASH framework. An acceptable and feasible handwashing station in our case required more than the presence of soap and water. Instead, attributes specific to the handwashing station technology, such as water storage capacity, durability, and maintenance requirements; contextual factors such as consistent access to water, the physical location of the technology within a household’s daily workflow; and psychosocial factors such as disgust, all contributed to how a handwashing station facilitated or inhibited handwashing at key times.

Ease of use the physical act of operating the handwashing station facilitates habit formation [22], and this proved an important technology-associated factor in our case. For women who maintain the home and care for children, adequate capacity was a major determinant in acceptability. Attributes specific to handwashing station technologies have been recognized in programmatic literature [13,23], and how the design of an acceptable handwashing station may take several iterations in order to address context-specific user requirements successfully [14]. This was the case in this study as well. However, in our view, behavioural models on water and sanitation do an imperfect job of acknowledging factors associated with the technology, and IBM-WASH [17] was especially useful in informing the full range of technology-related influential factors.

The development of the IBM-WASH was concurrent with this formative research in which we were able to develop operational definitions of factors and examine interactions between dimensions of the IBM-WASH. Some of the factors related to the technology and contextual dimensions were organized in the framework based on the data in this paper [17]. In that sense this formative research in-turn helped validate the IBM-WASH framework.

Regarding contextual factors, provision of a soapy water bottle without a water storage vessel helped to convert an existing tube well or hand pump to a handwashing station where the former provided reliable water access. However, this did not address the convenience of a second station at the food preparation area. It soon became evident that more than one handwashing stations might be required. This eventuality was not originally anticipated.

Psychosocial factors such as disgust towards faeces has been described in the literature as a motivator to handwash [4,24]. In this study, disgust towards faeces was found to be a barrier to food-related handwashing when the same handwashing station and soap was used for faeces-related handwashing. Furthermore, disgust was found to be a barrier to faeces-related handwashing if same tap, water, and soap were shared with and touched by toilet users from other households. Other psychosocial motivators such as knowledge and self-efficacy are acknowledged by many behavioural models [23,25], yet these motivators did not help distinguish between designs.

Physical attributes of the handwashing station included in the IBM-WASH and other models [23,24] are essential to understanding and promoting handwashing with soap because the behaviour is facilitated by specific products. For women who maintain the home and care for children, an adequate capacity was a major determinant in acceptability. Previous studies have demonstrated how access to water influences hygiene practices [26,27], and facilitates convenient access throughout the day. Though outside the scope of our intervention, we identified water access as an important community level factor. Most urban residents relied on electricity for water access and faced rolling blackouts each day. In this type of setting, the ability to store water may underlie the success or failure of a handwashing station. Expanding these findings to different contexts may also result in similar preference for water storage capacity. Involving women in the selection of an acceptable handwashing station in this study helped to identify some of the barriers to successful handwashing while underscoring the importance of water access and convenience to users [28].

Additionally, our findings suggest that handwashing station components must coexist with the surrounding household environment. Figueroa and Kincaid discuss how attitudes toward a product can positively or negatively influence use [29]. Furthermore, Devine found that a human-centred design process helped to identify barriers and facilitators to frequent use [14]. In this setting, we found an acceptable and feasible handwashing station must be delivered as a complete system. The kitchen bucket and 40 Litre bucket handwashing station designs included a stool and basin for this reason, and were highly acceptable.

We focused our analysis on factors occurring in the Habitual, Behavioural, Individual and Interpersonal/Household Levels of IBM-WASH for two reasons. First, Community and Societal/Structural factors shaping the feasibility and acceptability of handwashing station designs were already considered in selecting candidate designs. Second, the subsequent RCTs were to measure the effects of handwashing that occurs in the household, hence our formative research had the most direct relevance to the Behavioural, Individual, and Household Levels of our conceptual framework.

### Limitations

This research focused largely on the acceptability and feasibility of different handwashing station designs. Another study is necessary to measure the effectiveness of a chosen handwashing station on actual handwashing practice. Because handwashing stations were provided free of charge, there was likely a positive bias toward handwashing hardware, and in particular to the more costly designs. Likewise, the study did not include measures for desirability or market value. However, in the urban site, there were references to perceived product value, indicating that such measures would be beneficial. Specific questions targeting factors at the Community level, such as access to markets and resources and shared community values surrounding hygiene, may have provided greater context for selecting a handwashing station design for programmatic use, beyond a randomized trial.

## Conclusions

A number of contextual, psychosocial and technological factors influence use of handwashing stations at five aggregate levels, from habitual to societal. In interventions that require a handwashing station to facilitate frequent handwashing with soap, elements of the technology, such as capacity, durability and location(s) within the household are key to high feasibility and acceptability. Access to water, convenience, and disgust towards faeces-related handwashing stations may require more than one handwashing station per household.

Using constructs outlined in IBM-WASH may help identify key components of a behavioural intervention and key multi-level determinants influencing behaviour change in specific settings. Though the findings from this research were used to directly inform an intervention for subsequent research, this methodology may be used to develop successful interventions for both programmatic and research purposes.

## Competing interests

The authors declare that they have no competing interests.

## Authors’ contributions

KRSH conducted qualitative data analysis, the synthesis and interpretation of results and was the primary author of the final manuscript. PJW provided conceptual guidance for data analysis and manuscript development. PJW, EL, LU, FN, PK, AA and SL conceived of the study design and provided support for field trials and data collection. PJW, EL, RD and KRSH contributed to the development of the IBM-WASH-structured analytical approach. AA and NCD provided detailed information on study methods and lead the qualitative field research team. All authors provided feedback and read and approved the final manuscript.

## Pre-publication history

The pre-publication history for this paper can be accessed here:

http://www.biomedcentral.com/1471-2458/13/877/prepub
